# Evaluation of the Deki Reader™, an automated RDT reader and data management device, in a household survey setting in low malaria endemic southwestern Uganda

**DOI:** 10.1186/s12936-017-2094-3

**Published:** 2017-11-07

**Authors:** Caesar Oyet, Michelle E. Roh, Gertrude N. Kiwanuka, Patrick Orikiriza, Martina Wade, Sunil Parikh, Juliet Mwanga-Amumpaire, Yap Boum

**Affiliations:** 1 Mbarara University of Science and Technology, P.O. Box 1410, Mbarara, Uganda; 2 Yale School of Public Health, 60 College Street, New Haven, CT 06520 USA; 3 Epicentre Mbarara Research Centre, P.O. Box 1956, Mbarara, Uganda

**Keywords:** Deki Reader™, Malaria, Rapid diagnostic test, Malaria surveillance, Uganda

## Abstract

**Background:**

Early diagnosis of suspected malaria cases with a rapid diagnostic test (RDT) has been shown to be an effective malaria control tool used in many resource-constrained settings. However, poor quality control and quality assurance hinder the accurate reporting of malaria diagnoses. Recent use of a portable, battery operated RDT reader (Deki Reader™, Fio Corporation) has shown to have high agreement with visual inspection across diverse health centre settings, however evidence of its feasibility and usability during cross sectional surveys are limited. This study aimed to evaluate the performance of the Deki Reader™ in a cross-sectional survey of children from southwestern Uganda.

**Methods:**

A two-stage, stratified cluster sampling survey was conducted between July and October 2014 in three districts of southwestern Uganda, with varying malaria transmission intensities. A total of 566 children aged 6–59 months were included in the analysis. Blood samples were collected and tested for malaria using: the SD Bioline Malaria Ag Pf/Pan RDT and microscopy. Results were compared between visual inspection of the RDT and by the Deki Reader™. Diagnostic performance of both methods were compared to gold-standard microscopy.

**Results:**

The sensitivity and specificity of the Deki Reader™ was 94.1% (95% CI 69.2–99.6%) and 95.6% (95% CI 93.4–97.1%), respectively. The overall percent agreement between the Deki Reader™ and visual RDT inspection was 98.9% (95% CI 93.2–99.8), with kappa statistic of 0.92 (95% CI 0.85–0.98).

**Conclusions:**

The findings from this study suggest that the Deki Reader™ is comparable to visual inspection and performs well in detecting microscopy-positive *Plasmodium falciparum* cases in a household survey setting. However, the reader’s performance was highly dependent on ensuring adequate battery life and a work environment free of dirt particles.

## Background

Malaria imposes a substantial burden in a majority of sub-Saharan African countries. The widespread use of malaria rapid diagnostic testing (RDT) has been a critical step towards the prompt and accurate diagnosis of malaria [1–4]. Although the use of malaria RDTs has vastly improved malaria case management, the accurate and timely reporting of malaria cases for monitoring malaria trends remains a challenge in resource-constrained countries [5]. For malaria surveillance, most resource-limited countries rely on health facility records which are often incomplete and challenging to access [5, 6]. Collection of malaria data requires monthly reporting of cases from every health facility to a district record office where data is forwarded to the central record office. Delays in reporting impede timely decision-making for effective implementation of malaria control programmes [7].

To improve the accuracy of malaria diagnosis and reporting, a cell-phone based device, the Deki Reader™, has been introduced by Fionet Corporation. The Deki Reader™ has a workflow plan similar to a standard operating procedure which increases the likelihood that test procedures are adhered to for accurate diagnosis. Following processing of the RDT, the Deki Reader™ interprets and reports the results based on an analysis of the picture captured by the reader [8]. In addition to the test result, the patient’s demographic information, global positioning system (GPS) data of the testing site, testing personnel, date and time of testing can also be collected and all the information is transferred to a secured cloud database [9]. Data in the cloud database can be accessed by any authorized personnel and feedback can be administered in real-time. Such timely reporting can assist national malaria control programme officers in monitoring disease burden and targeting their efforts to high burden areas. Previous reports have shown the performance of the Deki Reader™ to have high agreement with visual malaria RDT inspection at the health facility level [8, 10, 11]. However, there have been no studies that have evaluated the performance of the Deki Reader™ in a household survey setting.

## Methods

### Study design and population

This sub-study was part of a cross sectional study aimed at assessing the prevalence of malaria parasitaemia among children 6–60 months of age across three districts of southwestern Uganda [12]. Sampling was performed between July and October 2014, during the low transmission season, in Bushenyi, Isingiro, and Mbarara district. Participants were sampled as previously described [12]. In brief, 20 villages from each district were stratified by their urban and rural status and randomly selected using the probability proportionate to the population size sampling [13]. The number of households selected from each village was weighted based on the population size of each district and households were randomly selected using the procedure of the World Health Organization (WHO) Extended Programme on Immunization (EPI) [14]. Only one child was selected per household. If more than one child met the inclusion criteria in a home, the participating child was randomly selected. All children whose parents provided parental consent were surveyed regardless of their current malaria status.

### Description of the Deki Reader™

The Deki Reader™ is a portable, battery-operated malaria RDT reader targeted for use by health workers. It is able to interpret a wide array of commercially-available malaria RDTs, provide workflow guidance, and capture and transmit point-of-care testing and patient demographic information over local mobile phone networks onto a secure cloud database [8, 9]. A portal provides access to web-based data analysis tools for principal investigators who are able to remotely monitor testing, review RDT images, and communicate feedback to the study team. The study investigators and research personnel of this study were trained in a 2-day workshop on how to operate the Deki Reader™. Alongside RDT results, data on GPS coordinates of the testing site, age and sex were collected on the device.

### Study procedures

Upon obtaining informed consent from the parent/guardian of the child, a short questionnaire was administered to the parent/guardian of the child to collect data on demographics and malaria control practices of the household. A trained laboratory technician collected approximately 250 μL (5 drops) of blood from each child by finger prick. Samples were used to detect malaria parasitaemia by blood smear and by RDT, using a combined *Plasmodium falciparum* histidine-rich protein-2 (HRP-2) and *Plasmodium* lactate dehydrogenase (pLDH) RDT (SD Bioline Malaria Ag Pf/Pan, Catalogue No. 05FK60, Standard Diagnostics Inc, Republic of Korea). RDT testing was performed according to the manufacturer instructions. The SD Bioline Malaria Ag Pf/Pan RDT detects both HRP-2 antigen specific to *P. falciparum* and pLDH exhibited by all *Plasmodium* species. A test result was only considered positive if the internal control and either the HRP-2 band and/or pLDH band(s) were positive. The RDT was immediately processed by the Deki Reader™ by a separate research staff technician so that both research technicians remained blinded to each RDT interpretation. Participants who were RDT positive by visual inspection and/or the Deki Reader™ were referred to the nearest health centre for case management.

### Microscopy diagnosis in the laboratory

For microscopy examination, thick and thin capillary blood smears were prepared as previously described [12]. Smears were stained with a 10% Giemsa solution (pH 7.2) for 15 min. Thick blood smears were used to detect parasite density, while thin films were examined to confirm *Plasmodium* species. A smear was declared negative if examinations using the 100 × oil immersion lens did not show any gametocytes or trophozoites [15]. Parasite density was calculated by counting the number of parasites against 200 leukocytes (or 500, if the count is < 10 parasites/200 leukocytes) multiplied by 8000, assuming 8000 WBCs/μL [16]. All smears were independently read by two microscopists who were blinded to the RDT results. A discordant result was defined as a difference of parasite counts of > 50 parasites/WBCs. Discordant results were resolved by a third reader [15].

For all patients with either a positive microscopy or RDT result, species confirmation was conducted using nested polymerase chain reaction (PCR) (Promega GoTaq Flexi DNA polymerase, Promega, Madison, WI) to detect either *P. falciparum, Plasmodium vivax, Plasmodium ovale, Plasmodium malariae,* and *Plasmodium knowlesi*. Primers were complementary to the *Plasmodium* small subunit ribosomal DNA gene as previously described by Singh et al. [17]. Positive controls were acquired from Malaria Research and Reference Reagent Resource Center (MR4, BEI Resources Repository, NIAID, USA).

### Statistical analysis

The data was analysed using STATA version 12.0 (StataCorp, College Station, TX, USA). Percentage of positive, negative, and overall agreement and Cohen’s kappa were used to measure inter-rater agreement between visual inspection and the Deki Reader™. Sensitivity and specificity were used to assess the diagnostic performance of the Deki Reader™ compared to the gold-standard, blood smear microscopy. McNemar’s test was used to compare differences in sensitivity and specificity between visual inspection and the Deki Reader™. P-values less than 0.05 were considered statistically significant.

### Ethics approval

The study was approved by the Institutional Research Committees of Mbarara University of Science and Technology, Uganda National Council for Science and Technology (Protocol Number HS 1684), and the Yale School of Public Health.

## Results

### Description of study population

Six-hundred and thirty-one children under 5 years of age were surveyed from July to October 2014. The mean age was 2.38 years (standard deviation 1.26 years) and comprised of 49% males (Table 1). Prevalence of malaria by microscopy was 3.5 and 6.7% by RDT using visual inspection [12]. Of the 631 children, data on 65 (10%) children were declared invalid by the Deki Reader™, with the message “RDT put too late”. These events occurred when the Deki Reader™ ran out of battery during the testing process and were, therefore, excluded from the analysis. Thus, the final sample size for this study consisted of 566 children. Four hundred and twelve (72.8%) children were sampled from rural villages. One hundred and twenty-seven (22.4%) children were recruited from Bushenyi district, 203 (35.9%) from Isingiro, and 236 (41.7%) from Mbarara. The proportion of blood-smear positive malaria cases was 3.9% in Bushenyi, 4.9% in Isingiro, 0.8% in Mbarara, and 3.0% across all districts. The proportion of RDT positive cases by visual inspection was 5.5% in Bushenyi, 13.8% in Isingiro, 1.3% in Mbarara, and 6.7% overall.

**Table 1:** Demographic characteristics of the study participants

Characteristic	Mbarara (n = 236)	Bushenyi (n = 127)	Isingiro (n = 203)	Total (N = 566)
Male (%)	112 (47.5)	61 (48.0)	105 (51.7)	278 (49.1)
Female (%)	124 (52.5)	66 (52.0)	94 (48.3)	288 (50.9)
Age (years), mean ± SD	2.38 ± 1.24	2.33 ± 1.20	2.41 ± 1.32	2.38 ± 1.26
Rural (%)	177 (75.0)	103 (81.1)	132 (65.0)	412 (72.8)
Urban (%)	59 (25.0)	24 (18.9)	71 (35.0)	154 (27.2)

### Timeliness of data reporting by the Deki Reader™

Seventy-four percent of the Deki Reader™ records were uploaded onto the cloud database within the first 24 h of the data collection, 92% reached the database within 48 h, and all records were available on the portal within 1 week of collection.

### Reliability of Deki Reader™ interpretation of RDT results compared to visual inspection

Of the 566 samples, the Deki Reader™ detected 40 (7.2%) positive cases compared to 38 (6.7%) by visual inspection. The Deki Reader™ recorded 30 positive results in rural areas as compared to 10 in urban areas (p value 0.8). The overall percent agreement between the Deki Reader™ and visual inspection was 98.9% (95% CI 93.2–99.8). Positive percent agreement was 94.7% (95% CI 82.3–99.4) and negative percent agreement was 99.2% (95% CI 98.1–99.8) (Table 2). The kappa agreement between visual malaria RDT interpretation and Deki Reader™ was 0.92 (95% CI 0.85–0.98) (Table 2). The overall performance agreement of Deki Reader™ between the rural and urban villages was 99.9% (95% CI 97.3–100%). Positive and negative percent performance agreement of the Deki Reader™ between rural and urban villages was 97.9% (95% CI 96.7–99.1%) and 98.8% (95% CI 97.2–99.6%), respectively.

**Table 2:** Comparison of SD Bioline Malaria Ag Pf/Pan RDT interpretation between the Deki Reader™ and visual inspection

	Visual inspection		Kappa (95% CI)	Percent agreement (95% CI)		
	Positive	Negative		Positive	Negative	Overall
Deki Reader™						
Positive	36	4	0.92 (0.85–0.98)	94.7 (82.3–99.4)	99.2 (98.1–99.8)	98.9 (92.3–99.8)
Negative	2	524				

### Diagnostic performance of visual inspection of RDT against microscopy

Table 3 presents the diagnostic performance of visual inspection of the SD Bioline Malaria Ag Pf/Pan RDT compared to microscopy. Of the 566 samples tested, 17 samples were microscopy-positive compared to 38 that were found positive by RDT. Fifteen samples were positive by both diagnostic methods and 25 samples were discordant between the two methods. Two samples were falsely negative by RDT, resulting in 88.2% (95% CI 63.7–98.5) sensitivity. Parasite density of these two samples was 53 parasites/μL and 109 parasites/μL. Twenty-three samples were falsely positive by visual inspection, making specificity 95.8% (95% CI 93.8–97.3).

**Table 3:** Performance of visual interpretation and Deki Reader™ of the SD Bioline Malaria Ag Pf/Pan RDT against microscopy

	Microscopy		Sensitivity (95% CI)	p-value^*^	Specificity (95% CI)	p-value^*^
	Positive	Negative				
Visual interpretation						
Positive	15	23	88.2 (63.7, 98.5)		95.8 (93.8, 97.3)	
Negative	2	526		0.4		0.06
Deki Reader™						
Positive	16	24	94.1 (73.0, 99.0)		95.5 (93.4, 96.9)	
Negative	1	525				

^*^Reported p-value using McNemar’s test to assess difference in sensitivity between visual inspection and the Deki Reader™

### Performance of Deki Reader™ and visual interpretation against microscopy

Forty (7.1%) samples were considered positive by the Deki Reader™ compared to 17 (3.0%) based on microscopy (Table 3). Twenty-five samples were discordant between results read from the Deki Reader™ and microscopy; however, the Deki Reader™ only produced one false negative result. Twenty-four samples were false positives by the Deki Reader™ when compared to microscopy, though, 13/24 (54.2%) turned out to be PCR-positive. Sensitivity and specificity of the Deki Reader™ was 94.1% (95% CI 73.0–98.9) and 95.8% (95% CI 93.8–97.2), respectively. Differences in sensitivity and specificity values between visual inspection and the Deki Reader™ were not statistically significant (p = 0.40 and p = 0.06, respectively). Seven of the samples were repeated when trained field laboratory personnel found dirt particles lodged into the result window of the RDT and the reader initially detected the dirty RDTs as the “wrong RDT”.

## Discussion

This study aimed to evaluate the accuracy and reliability of the Deki Reader™ when used in a household survey setting. Compared to visual inspection of the SD Bioline Malaria Ag Pf/Pan RDT, the results given by the Deki Reader™ showed almost perfect agreement (kappa > 80%), according to Landis and Koch’s criteria for inter-rater reliability [18–20], and no significant differences in sensitivity and specificity. Findings from this study are consistent with studies conducted in rural Tanzania and Columbia which obtained similar agreement values [8] and an in vitro study that demonstrated the limit of detection of the Deki Reader™ to be similar to that of microscopy (approximately 20 parasites/µl) [21].

The Deki Reader™ was able to identify one additional microscopy-positive case that was undetectable by visual inspection, thereby resulting in a slightly higher sensitivity value compared to visual inspection (94.1% vs. 88.2%), though this difference was not statistically significant (p = 0.70). Though the current study however was not powered to detect a difference in sensitivities between the visual inspection and Deki Reader™, our findings are consistent with previous studies which have shown the Deki Reader™ to demonstrate high diagnostic performance, comparable to that of visual inspection of RDT by trained personnel [9, 10, 21, 22].

In addition to its high diagnostic performance, the Deki Reader™ has shown to be beneficial in other ways. It can accurately collect information on global positioning system (GPS) coordinates of the testing site, patient demographics, and the time and date of testing. This information is securely stored onto a cloud database which can be accessed directly by authorized personnel at any time [21], suggesting the Deki Reader™ can be a practical tool for malaria surveillance. The Deki Reader™ did not show variation in performance when used in the rural villages and urban villages (Table 2), and network connectivity did not affect the performance of the reader.

The Deki Reader™ did exhibit a few limitations. The timeliness of reporting was highly dependent on cell phone receptivity and the strength of connection. Though 74% of the data was uploaded on the same day, when sampling occurred in villages with sporadic or no cell-phone network access, data was not uploaded onto the portal until the reader was in transported to an area of established network connectivity. Thus, if the Deki Reader™ is to be used in remote settings, timely data reporting to the central site may require transporting the reader to a network receptive area where data can be readily uploaded. Additionally, 10% of the samples resulted in failed readings by the Deki Reader™, the majority of which were considered to be due to low battery power. The device needed to be recharged every 5 days to avoid invalid results and this will need to be considered for long field visits to areas little to no access to electricity. Lastly, seven samples had to be repeated when dirt particles were lodged into the result window of the RDT. Though it is not certain that dirt was the causal reason for the invalid processing error, the RDT and corresponding work station should be kept clean to maintain high reader performance, which was challenging in field, particularly during the rainy season.

## Conclusions

The results of this study provide further evidence toward the feasible use of the Deki Reader™ across varied health settings. The present findings suggest that the Deki Reader™ is comparable to visual inspection of RDTs and performs well in detecting microscopy-positive *P. falciparum* cases. In addition to accurately recording diagnostic information, epidemiological data was readily collected and uploaded onto the database within a week, demonstrating the reader’s potential role in strengthening malaria surveillance, especially in remote areas. In large scale household cross sectional surveys, such as the Malaria Indicator Survey, the Deki Reader™ may be an attractive tool to increase accurate and complete data collection and reduce the need for additional equipment in the field, including paper-based case report forms and GPS receivers. However, extra care must be taken to maintain the integrity of the RDT membrane prior to testing.
